# ComPath: comparative enzyme analysis and annotation in pathway/subsystem contexts

**DOI:** 10.1186/1471-2105-9-145

**Published:** 2008-03-06

**Authors:** Kwangmin Choi, Sun Kim

**Affiliations:** 1 School of Informatics, Indiana University, Bloomington, IN 47408, USA; 2 Center for Genomics and Bioinformatics, Indiana University, Bloomington, IN 47408, USA

## Abstract

**Background:**

Once a new genome is sequenced, one of the important questions is to determine the presence and absence of biological pathways. Analysis of biological pathways in a genome is a complicated task since a number of biological entities are involved in pathways and biological pathways in different organisms are not identical. Computational pathway identification and analysis thus involves a number of computational tools and databases and typically done in comparison with pathways in other organisms. This computational requirement is much beyond the capability of biologists, so information systems for reconstructing, annotating, and analyzing biological pathways are much needed. We introduce a new comparative pathway analysis workbench, ComPath, which integrates various resources and computational tools using an interactive spreadsheet-style web interface for reliable pathway analyses.

**Results:**

ComPath allows users to compare biological pathways in multiple genomes using a spreadsheet style web interface where various sequence-based analysis can be performed either to compare enzymes (e.g. sequence clustering) and pathways (e.g. pathway hole identification), to search a genome for *de novo *prediction of enzymes, or to annotate a genome in comparison with reference genomes of choice. To fill in pathway holes or make *de novo *enzyme predictions, multiple computational methods such as FASTA, Whole-HMM, CSR-HMM (a method of our own introduced in this paper), and PDB-domain search are integrated in ComPath. Our experiments show that FASTA and CSR-HMM search methods generally outperform Whole-HMM and PDB-domain search methods in terms of sensitivity, but FASTA search performs poorly in terms of specificity, detecting more false positive as E-value cutoff increases. Overall, CSR-HMM search method performs best in terms of both sensitivity and specificity. Gene neighborhood and pathway neighborhood (global network) visualization tools can be used to get context information that is complementary to conventional KEGG map representation.

**Conclusion:**

ComPath is an interactive workbench for pathway reconstruction, annotation, and analysis where experts can perform various sequence, domain, context analysis, using an intuitive and interactive spreadsheet-style interface.

## Background

Comparative analysis of multiple genomes has become a very important research method as more genome sequences become available. Biological pathway analysis is also typically performed in comparison with multiple genomes of choice, using a number of computational tools and databases. A biological pathway involves a number of enzymes and its substrates and products. In addition, pathways interact with each other. Thus comparative analysis of pathways is quite complicated and can hardly be done without well designed pathway analysis software systems. A number of automated pathway comparison systems have been developed: The SEED [1], Pathway Tools [2], KAAS (KEGG Automatic Annotation Server) [3], KOBAS (KEGG Orthology-Based Annotation System) [4], Path-A (Pathway Analyst) [5], TIGR Comprehensive Microbial Resources [6], and JGI Integrated Microbial Genomes [7].

The SEED provides a web-based comparative genome annotation environment based on 'subsystems.' Subsystems are a set of functional roles found in any common biological process including metabolic pathways, phenotypes, or multi-subunit complex structures. Subsystems in multiple genomes are conveniently represented in a table format: functional roles (i.e. subsystems) are represented in columns, genomes represented in rows, and cells are populated with the genes having corresponding function. A populated subsystem in the table specifies which genomes include gene variants of the subsystem. This populated subsystem approach is an intuitive way to visualize pathway components of multiple genomes. Detection of subsystems in a large number of genomes is not trivial, thus a computational method is developed to detect subsystems automatically [8].

Pathway Tools uses the PathoLogic algorithm to determine enzymatic reactions catalyzed by each gene product in a query genome and then match detected reaction list against all available pathways from a reference database. PathoLogic accepts sequences of a fully annotated genome from GenBank [9] and MetaCyc [10] is used as a reference pathway database. PathoLogic uses the annotation information from GenBank, as opposed to sequence similarity information used in other systems, and the EC assignment as evidence for the presence of each pathway in the reference database for the genome of interest. Once the matching step is complete, PathoLogic infers a set of reactions expected to occur in the target genome and determines which of those pathways are likely to exist in the target genome.

KAAS and KOBAS are systems to annotate input protein sequences with KO (KEGG Ontology). KAAS provides functional annotation of genes by BLAST comparisons, single best hit (SBH) and bidirectional best hits (BBH), against the manually curated KEGG/GENES database [11,12]. KO assignments to genes and predicted KEGG pathways are generated as output. KOBAS provides statistical significance tests for predicted pathways. KO terms are assigned based either on sequence similarity to entries in KEGG/GENES or on cross-database links in KEGG/GENES when a list of sequence identifiers is available in the databases. After KO assignment, frequently occurring or statistically significantly enriched pathways of the query sequences are identified in comparison with the background model.

Path-A (Pathway Analyst) takes a set of query protein sequences from a genome and identifies which sequences are likely to exist in any of its supported model pathways using several sequence analysis techniques (e.g. SVM, BLAST and HMM). The model pathway approach enables the pathway prediction algorithm to predict instances of a pathway with variations in pathway structure that were never observed in the training pathway set. Path-A currently provides abstract models for 10 pathways, spanning 125 genome-specific pathway instances.

Comprehensive Microbial Resources (CMR) at TIGR allows users to access all bacterial genome sequences completed to date. CMR provides two types of annotation resources: primary annotation from the genome sequencing center and TIGR annotation that is generated by an automated annotation process at TIGR. The CMR Pathway Tool kit consists of three categories of pathway analysis tools: "Genome Properties" provides information on the characteristics of organisms derived from genomic data and literature sources; "Genome Properties Detailed Comparison" provides detailed step information for a set of genomes users select; and "KEGG Pathway Display" highlights KEGG's pathway steps based on the presence or absence of EC evidence in the CMR.

Integrated Microbial Genomes (IMG) at JGI provides comparative analysis of microbial genomes in an integrated genome context. The data model underlying the IMG system incorporates primary genomic sequence information, computationally predicted and curated gene models, pre-computed sequence similarity data, functional annotation, and pathway information. Microbial genome data analysis in IMG is performed in the comparative context of multiple microbial genomes where a number of tools can be used to compare genomes in terms of organism-specific statistics, genes and sequence conservation.

Since pathway analysis is quite complicated involving a number of tools and databases, no single system is better than others. Users should choose a system that fits to their research interests. In this paper, we introduce a new web-based comparative pathway workbench system, ComPath, where users can conveniently perform various analyses for common biological pathways among multiple genomes of their choice. Table 1 provides comparison of ComPath and five existing systems. Below we emphasize following features of our system in comparison with existing systems.

**Table 1:** Comparison to related works. ComPath is compared with five existing pathway analysis systems: The SEED, Pathway Tools, KAAS, KOBAS, and Path-A.

**System**	**ComPath**	**The SEED**	**Pathway Tools**	**KASS**	**KOBAS**	**Path-A**
**Focus**	More interactive interface, more data integration	Annotation by EC, RC, and GO	Genomic data integration, pathway prediction and annotation	Simple gene annotation using KO	Annotation by KO with statistical evaluation	Annotation against model pathway
**Reference Pathway Database**	KEGG	Subsystems	*De novo *generation by PathoLogic algorithm	KEGG	KEGG	10 model pathways – spanning 125 organism-specific pathway instances
**Classifier**	FASTA Whole-HMM CSR-HMM PDB-domain search	BLAST	PathoLogic	BLAST-BBH BLAST-SBH	BLAST	Opt HMM BLAST-HMM BLAST Motif SVM HMM
**Ontology**	EC, GO	EC, RC, GO	Pathway Tools Ontology, EC	KO	KO	EC
**Sequence analysis**	CDD search, Prosite pattern search, phylogenetic tree analysis, BAG sequence clustering, Gibbs motif sampling, iGibbs	Gene cluster search, Phylogenetic tree analysis	No	No	No	No
**Pathway representaion**	Automatic generation based on information in KEGG/KGML	KEGG map	Automatic generation based on MetaCyc database	KEGG map	KEGG map	KEGG map
**Genome context**	CGView, Gene cluster search	GBrowse, Gene cluster search	Genome browser at BioCyc site	No	No	No
**Data management**	Yes	Yes	Yes	Yes	Yes	Yes

▪ **Easy-to-use interface**: ComPath provides an easy-to-use, interactive pathway analysis and annotation environment/workbench by integrating multiple data sources and analysis tools into a single framework. ComPath represents biological pathways on a spreadsheet-style interface as the SEED system does, but our spreadsheet is designed to be more interactive so that users can directly perform sequence, motif, and context analyses on this spreadsheet.

▪ **Flexible pathway assignment**: ComPath provides a total of 327 model pathways combining both KEGG and The SEED: 205 pathways from KEGG database and 122 subsystems from The SEED.

▪ **Sequence/motif analysis-oriented**: ComPath uses structural domain and context information for pathway reconstruction, in addition to sequence homology information.

▪ **Prediction and evaluation**: ComPath, KAAS and KOBAS allow annotation of a whole genome in the pathway context. ComPath provides a suite of computational methods that can be used to verify predicted pathway components using sequence/motif/phylogeny analysis tools while KAAS and KOBAS allow use of standard sequence alignment method (BLAST) only.

▪ **Genome context analysis**: ComPath provides tools for gene cluster detection and visualization based on sequence similarity and position information.

▪ **Pathway context analysis**: Users can explore enzyme relationships in terms of global pathway networks of EC, GO (Gene Onology), and RxID (reaction ID), not just in terms of predefined pathways such as those in KEGG. This allows users to explore relationships among different predefined pathways with respect to an enzyme of choice.

## Implementation

### System Overview

**Figure 1:**
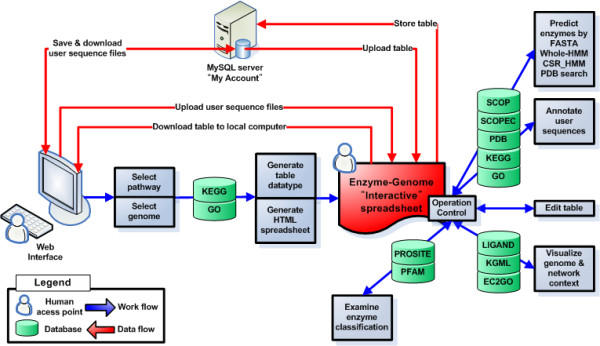
**The overall architecture of ComPath system: workflow and dataflow**. ComPath integrates various databases and analysis tools in the interactive spreadsheet. Users are able to perform sequence/context analysis, edit table, or upload/download table data type to "My Account" or desktop simply by manipulating this spreadsheet interface.

The ComPath system architecture is described in Figure 1. ComPath uses the KEGG database suite as a primary data resource: PATHWAY, GENES, LIGAND, and BRITE, for pathways, sequences, compounds/reactions and functional classification, respectively. In addition, sequence, structure, and domain databases are integrated into the system. Motif and structural domain information are retrieved from PFAM [13], PROSITE [14], SCOP [15], SCOPEC [16], SUPERFAMILY [17], and PDB database [18]. SCOPEC is used to map EC classification to structural domain information from SCOP. Full-length sequence information comes from Swiss-Prot and KEGG/GENES database. Mapping information of GO to EC, PFAM, and PROSITE are downloaded from the Gene Ontology site [19]. All these databases are internally integrated and URL links to CDD [20], GenBank [9], and UniProt [21] are provided on an interactive spreadsheet. In addition, a number of computational tools are integrated with data resources in a single unified framework.

Upon selection of a biological pathway and a set of genomes, users can perform the following tasks using the interactive enzyme-genome spreadsheet:

**1**. Enzyme sequences with the same EC category can be compared using various sequence- and structure-based computational tools.

**2**. Candidates for missing enzymes in particular genomes can be predicted and they can be further verified using computational tools. In this way, pathway holes may be filled in.

**3**. Un-annotated genomes can be easily compared against enzyme sequences in already well-annotated genomes in terms of a pathway of choice.

Detailed description of each step can be found in "The workflow" section. Below, we summarize important features of ComPath.

### Feature 1: Interactive Spreadsheet for pathway data manipulation and analysis (see Figure 1 and Figure 2)

**Figure 2:**
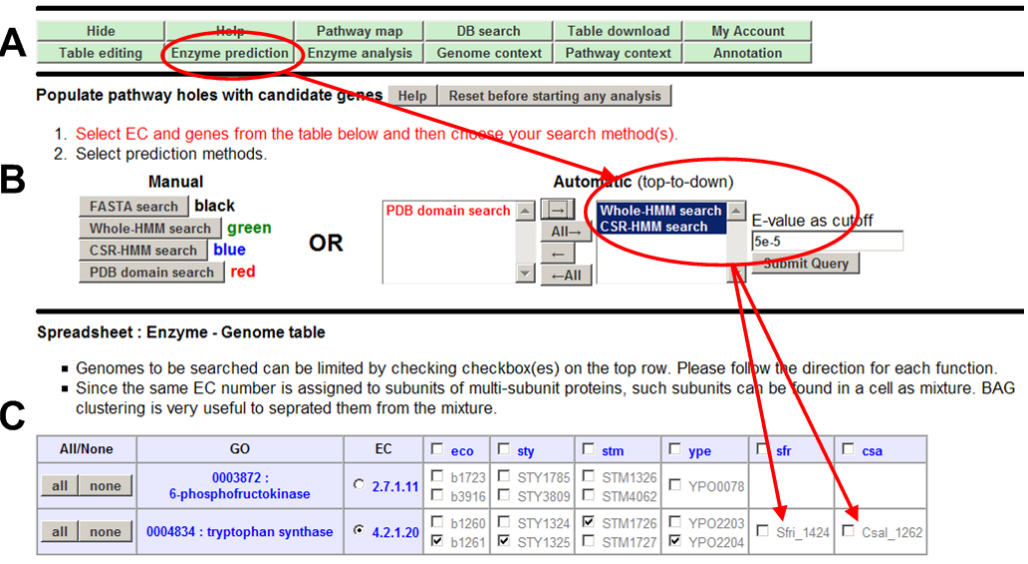
**ComPath's interactive spreadsheet**. Buttons in top panel (A) provide various functions to edit spreadsheet ("Table editing" button), predict pathway holes ("Enzyme prediction", B), evaluate candidate enzymes ("Enzyme analysis"), visualize genome/pathway context ("Genome context" and "Pathway context"), and annotate a given pathway of newly sequenced genome ("Annotation"). Panel B displays a list of methods for "Enzyme prediction." After choosing search methods in panel B, users are free to select any genes/genomes of interest in Enzyme-Genome table.

Users interact with the ComPath system using a spreadsheet style interface called "Table Data Type" [22]. Unlike many other systems, users are able to perform various sequence and network analyses on the web simply by manipulating the interactive spreadsheet (See Figure 2). Computational analysis functions are grouped into five categories, denoted by buttons on the web: (i) "Enzyme Prediction" for predicting *de novo *enzymes, (ii) "Enzyme analysis" for comparing/verifying enzyme predictions, (iii) "Genome context" for searching and visualizing genomic context of a selected enzyme (iv) "Pathway context" for visualizing network context of enzymes, reactions, and GO terms and (v) "Annotation" for automatically annotating genes in a newly uploaded genome in terms of KEGG pathways. Users can either download the current spreadsheet (table) into a local computer or store it into a MySQL server at any time by clicking "Table download" or "My Account" buttons. Tables that are stored or downloaded can be uploaded to the server anytime to resume pathway analysis. Each function will be explained in "Workflow" section in detail.

### Feature 2: Use of multiple enzyme prediction methods (see Figure 2)

One major goal of ComPath is to provide biologists with an exploratory computational environment/workbench to search for missing enzymes involved in a given biological pathway. To fill in pathway-holes, biologists can use sophisticated analysis methods in addition to standard sequence similarity methods such as BLAST [23] and FASTA [24]. Sequence similarity-based comparison methods (e.g. BLAST and FASTA) generally work well, but they may detect many false positives and often fail to determine functions for many genes. As a result, 20–60% of the proteins in most sequenced genomes still remain as "hypothetical" [25].

To handle this problem, it is necessary to use computational tools other than sequence similarity-based methods. ComPath provides motif- and structural-domain-based search tools in addition to standard similarity-based gene search. Enzyme search methods used in ComPath are (i) Whole-HMM search, (ii) CSR-HMM search, a novel method of our own, (iii) PDB-domain search, and (iv) FASTA search. The Whole-HMM method builds a profile HMM model using "whole" enzyme sequences that belong to the same EC group and searches query genomes with the HMM model. In contrast, the CSR-HMM method uses "common shared region" that is automatically computed by the BAG clustering algorithm [26] to build profile HMM models. The PDB-domain search method first searches SCOPEC database to retrieve SCOP-to-EC mapping information. SCOPEC is a database of catalytic domains that combines structural domain information from SCOP, full-length sequence information from SwissProt, and verified functional information from the Enzyme Classification (EC) database. Once SCOP IDs are collected, the second step is searching ASTRAL SCOP domain sequence database (based on PDB SEQRES records) or SUPERFAMILY sequences (default setting). SUPERFAMILY sequences are those with longer than 30 residues (20 in ASTRAL) and shorter sequences that are parts of other sequences are removed when filtering on sequence identity. If multiple sequences are collected, CSR-HMM search is done against target genome(s). If only one sequence is retrieved, FASTA search is performed instead. The FASTA search with whole enzyme sequences is also available, but this is not recommended for enzyme search due to its low specificity; see "Empirical evaluation of enzyme prediction methods" section.

Users can evaluate predicted enzyme candidates (see Step3 in "Workflow" section). This step is necessary since predicted candidates are often false positives. For example, inspection on the existence of common CDD [20] domains or PROSITE [14] patterns of a given EC group can be used as an evidence to suspect enzyme candidates as false positives. This type of dynamic analysis can be performed easily in ComPath.

### Feature 3: Physical context/gene neighborhoods analysis (see Figure 3)

**Figure 3:**
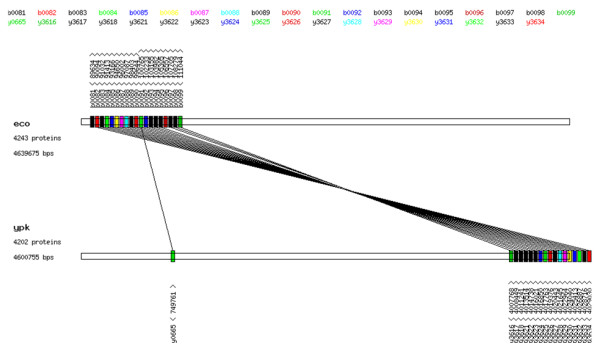
**Genome context analysis**. Two gene clusters are well conserved between *Escherichia coli K-12 MG1655 *and *Yersinia pestis KIM*. Genes are assigned with color according to corresponding EC category. Genes in black color are proteins with no EC assignment (generally, they are "hypothetical" proteins). Genome context was generated using our in-house physical context viewer integrated with the ComPath system.

ComPath provides two tools for visualizing gene neighborhood: an in-house physical context viewer (see Figure 3) and CGView for detecting and visualizing gene neighborhoods. The gene neighborhood, or physical context, is defined by setting a sequence similarity score cutoff for matching genes in different genomes and intergenic distance as a constraint. In a visualization of gene context using physical context viewer, genes are visualized with color scheme according to the EC category. Only two genomes can be compared in the current implementation. In addition, users can use the CGView genome browser [27] to navigate/visualize all enzymes in a genome and their positions. Physical context analysis is currently available only for prokaryotic genomes.

### Feature 4: Global pathway context/network neighborhood analysis (see Figure 4)

**Figure 4:**
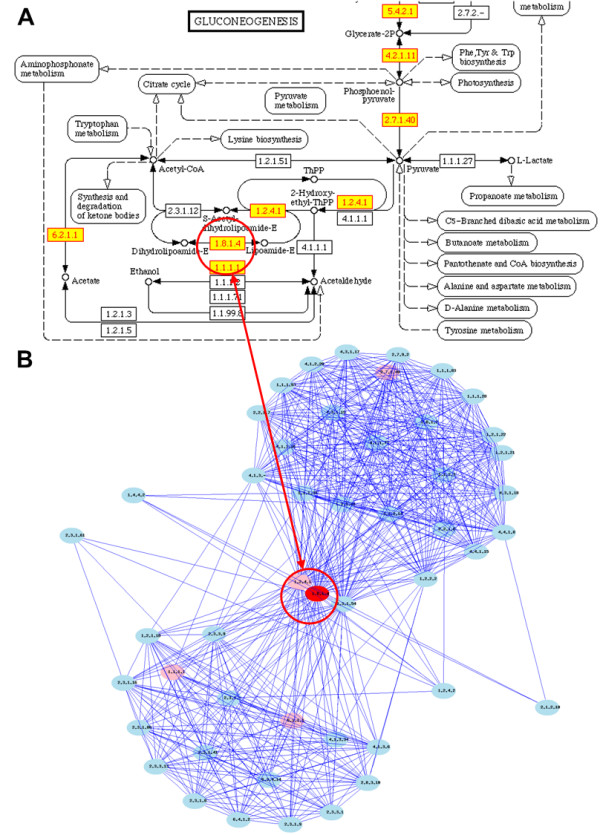
**Pathway context analysis**. A KEGG map uses artificially-dissected biological pathways, not global pathway. ComPath creates a global pathway network (the union of all pathways) for each genome. When a core node (red) and path length (or range) are specified as input by a user, neighboring nodes (pink and blue) are searched and visualized. Red and pink nodes belong to the same pathway; however blue nodes belong to different pathways.

The KEGG pathway map that we are utilizing in ComPath is a useful and convenient dissect of complex biological networks. However, there are two limitations in the pathway representation. First, each KEGG pathway is a union of all enzymes from all species participating in a given pathway theme (e.g. "Glycolysis") rather than "pathway" in a specific genome. Because of this, many reactions are often not coupled or connected in KEGG maps of a given genome. In addition, each KEGG pathway map is only a subset of global pathway network where numerous cross-talks among sub-networks exist. When the relationship is visualized, we can observe genes involved in the same functional theme are grouped as a module in a network of genes [28]. In general, these modules are not consistent with pre-defined pathways maps (e.g. KEGG maps) and enzymes are often involved in more than one pathway.

The table-type pathway representation in ComPath that is based on KEGG map is not very effective in showing pathway interactions in the global network. Thus ComPath dynamically generates a genome-specific directed/undirected graph of a given biological network by parsing KEGG/KGML database that provides catalytic reaction information including substrate(s), product(s), reaction ID(s), and directionality (reversible, irreversible) of a enzyme reaction. The original representation of a given catalytic network in KEGG/KGML is a "directed metabolite graph." Its nodes are metabolites and edges are reaction IDs (i.e. RxID). Starting from this metabolite graph, ComPath creates Rx graph, EC graph, and GO graph, where nodes are reaction IDs, EC numbers, and GO terms, respectively. The conversion from the metabolite graph to the Rx graph is the same as described in a recent paper [29]. EC graph is generated based on the Rx graph and RxID-EC relationship in KEGG/LIGAND database and the GO graph is derived from the EC graph by referring to EC2GO database. Due to the limitation of computational resources, ComPath visualizes only a sub-network of a given radius, a parameter the user can set a value to, from an enzyme which users are interested in. We recommend users to use Pajek [30] for detailed and faster global network analysis. ComPath generates Pajek input files of directed/undirected graph. In the current implementation, this feature only focuses on metabolic networks, but will be expanded to other biological networks.

### Feature 5: Genome annotation

ComPath can be used to automatically generate a tentative annotation of a whole genome in terms of model pathways. Candidate enzymes in a new genome that are not in the KEGG database are matched to enzymes in model pathways and a new table is created by filling entries corresponding to known enzymes in the model pathway context. In this way, the new table can help users understand how likely the pathway or subsystem exists in the new genome since known KEGG pathways or subsystems in SEED provide meaningful context for potential gene matches. Users can use three different ways to define pathways and subsystems: KEGG pathways, SEED subsystems, and user-defined EC sets.

When an un-annotated or poorly annotated genome in FASTA format is uploaded onto the interactive spreadsheet, ComPath performs automatic enzyme candidate searches using FASTA or CSR-HMM methods. Once cells in the spreadsheet are populated with candidate enzymes, users can start to perform enzyme comparison analyses as described in the previous sections and the result can be used as evidence for gene function determination. Users should carefully select reference genomes that are phylogenetically close to the genome being annotated. In the current web implementation, only one genome is accepted for genome annotation.

### Feature 6: Data management

The table can be easily edited, updated, and reloaded. At any time of pathway analysis, the table can be saved into either a local computer by downloading it or the MySQL data management server by clicking "My Account" button. To use the MySQL server, users need to register and get a user ID and password. Stored tables can be easily uploaded to the ComPath server and then the table at the time of the previous analysis can be re-generated by a single click on the web. In addition to the table, FASTA-format protein sequences can be stored in the MySQL database and retrieved later for annotation.

### The workflow

#### Step 1: EC-based pathway reconstruction (a spreadsheet table generation)

**Figure 5:**
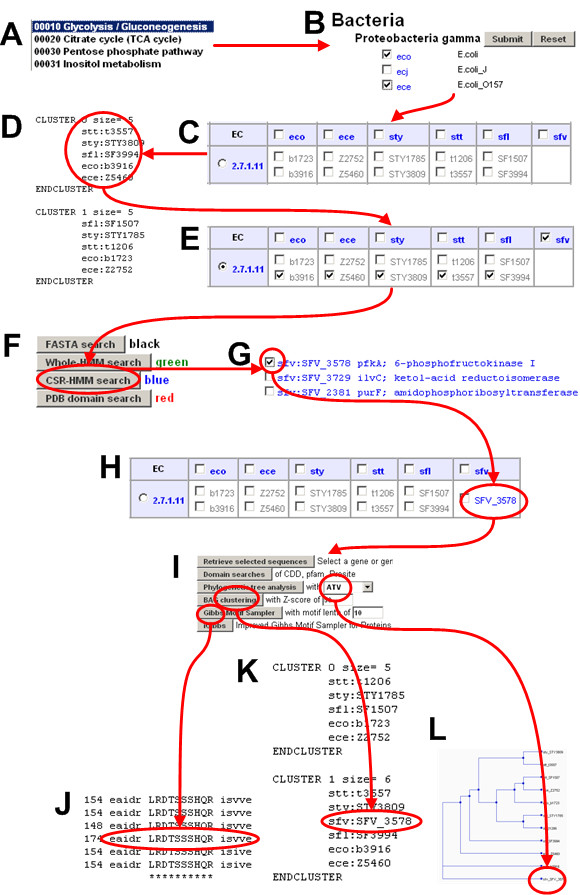
**A simple example of how ComPath works**. Glycolysis/glucogenesis pathway (A) and six gamma-proteobacteria species were selected (B). Six genomes are *Escherichia coli K-12 MG1655 *(eco), *Escherichia coli O157 EDL933 *(ece), *Salmonella typhi CT18 *(sty), *Salmonella enterica serovar typhi Ty*2 (stt), *Shigella flexneri 301 *(sfl), *Shigella flexneri 8401 *(sfv). A possible pathway hole was found in *Shigella flexneri 8401 *(C). To fill in this hole, query genes were carefully chosen based on BAG clustering (D) and target genomes were selected based on clustering result (E). A candidate gene detected by CSR-HMM method was added to the table (F-H) and confirmed using three different refinement methods: Gibbs motif sampler (J), BAG clustering (K), and phylogenetic tree analysis (L). See also Figure 7.

First, users need to select a pathway (Figure 5A) from the pre-defined KEGG pathway list or subsystems in The SEED and then select genomes from the genome taxonomy tree (Figure 5B) from 415 prokaryotic and eukaryotic genomes in KEGG. A union of EC categories that belong to a given biological pathway was pre-compiled from KEGG database. Users are recommended to select phylogenetically-close species for annotation purpose unless the goal is to trace the evolution of a metabolic pathway among relatively distantly-related genomes.

In case a new genome that is not among the genomes in KEGG is uploaded, ComPath searches the genome for candidate enzyme matches against selected genomes and fills in entries in the table with matches found. This step is called "EC-based pathway reconstruction." Detected genes are considered as candidates for pathway components. These candidates may need to be further verified (see Step 3 "Evaluation of enzyme classification"). Upon selection of a pathway and genomes, an interactive enzyme-genome spreadsheet, i.e. a table, is created on the web.

### Step 2: Identification of missing enzyme candidates

Users can perform the missing gene search as follows. (i) Select an EC number, a genome, and query genes in the table based on BAG clustering (see Figure 5C/D/E) or other prior knowledge, (ii) Select computational methods for enzyme search (see Figure 5F), and (iii) Select which candidate matches from the search result will be added to the table (see Figure 5G and 5H). Figure 5I/J/K/L show prediction refinement step using Gibbs motif sampler (see Figure 5J), BAG clustering (see Figure 5K), and phylogenetic tree analysis (see Figure 5L).

Although the EC system effectively describes biochemical functions for most of known enzymes, it has several problems. For example, a simple EC-based pathway reconstruction tends to miss true enzymes because the number of "hypothetical" proteins without EC assignment continues to increase. In addition, the EC system was developed before sequence and structural information of enzymes was available, so it is not designed to match catalytic function to enzyme structure in terms of family and superfamily of homologous proteins [31]. Thus, decisions on the pathway reconstruction and enzyme prediction using selected query sequences should be often guided by experts. We found that BAG clustering or other sequence classification methods (e.g. phylogenetic tree analysis) play an important role as shown in Figure 5D and the following two cases.

#### Case 1: Multi-subunits of enzyme complex

**Figure 6:**
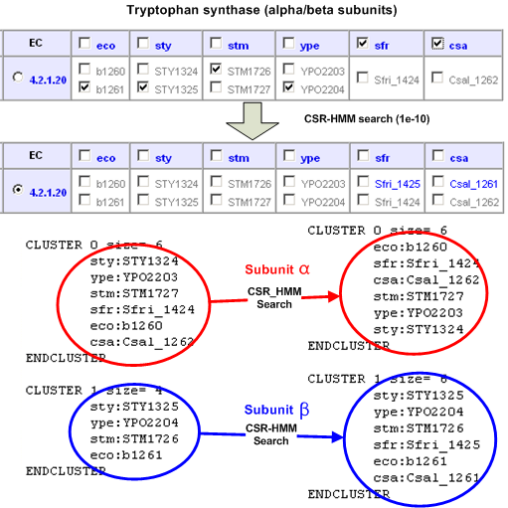
**Case I: multi-subunit enzyme complexes**. EC 4.2.1.20 and six gamma-proteobacteria species were selected: *Escherichia coli K-12 MG1655 *(eco), *Salmonella typhi CT18 *(sty), *Salmonella typhimurium LT2 *(stm), Yersinia pestis CO92 (ype) *Shewanella frigidimarina *(sfr), *Chromohalobacter salexigens *(csa). Initial pathway reconstruction detected only alpha subunits in *Shewanella frigidimarina *and *Chromohalobacter salexigens*. BAG separated alpha subunits from beta subunits clearly. After CSR-HMM search using beta subunits proteins as queries, two candidate beta subunit proteins are detected (Sfri_1425 in *Shewanella frigidimarina *and Csal_1261 in *Chromohalobacter salexigens*).

The enzyme subunits of a multi-subunit complex require more careful handling because they are grouped into the same EC category but do not have sequence similarity between different subunits. For example, tryptophan synthase (EC:4.2.1.20), catalyzing the last step in the biosynthesis of tryptophan, needs two separate subunits (proteins) to perform the catalytic function. Each of the two subunits exists as separate genes in bacteria and plants (alpha and beta subunits). The alpha chain is for the aldol cleavage of indoleglycerol phosphate to indole and glyceraldehyde 3-phosphate and the beta chain is for the synthesis of tryptophan from indole and serine. In Figure 6, initial pathway reconstruction only detects alpha subunits in *Shewanella frigidimarina *and *Chromohalobacter salexigens *and the BAG clustering result shows that alpha subunits and beta subunits can be separated into two clusters. After CSR-HMM search using beta subunit proteins as queries, two beta subunit proteins are detected (Sfri_1425 in *Shewanella frigidimarina *and Csal_1261 in *Chromohalobacter salexigens*). Again, BAG clustering shows that they are tryptophan synthase beta subunits.

#### Case 2: Isozymes

**Figure 7:**
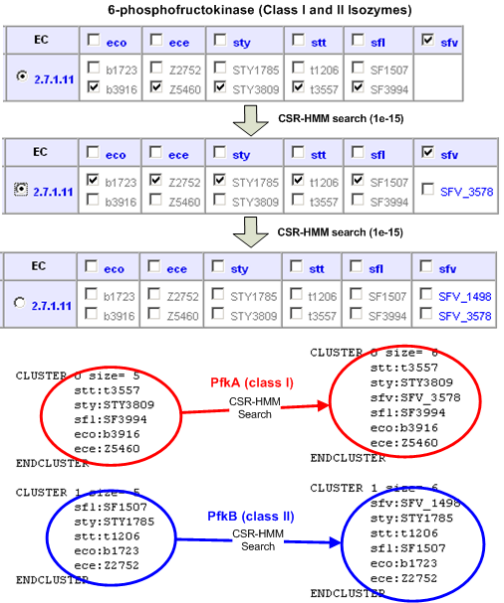
**Case II: isozymes**. EC 2.7.1.11 and six gamma-proteobacteria species were selected: Escherichia coli K-12 MG1655 (eco), Escherichia coli O157 EDL933 (ece), Salmonella typhi CT18 (sty), Salmonella enterica serovar typhi Ty2 (stt), Shigella flexneri 301 (sfl), Shigella flexneri 8401 (sfv). Initial pathway reconstruction detected no phophofructokinase gene in Shigella flexneri 8401 (sfv). BAG clustering clearly divides class I and class II isozymes from five species. To detect two isozymes classes in Shigella flexneri 8401, two sequential CSR-HMM searches using two set of protein sequences as queries detect SFV_3578 (class I) and SFV_1498 (class II).

Isozymes are enzymes with the same catalytic activity, but they are distant in terms of sequence similarity. For example, phosphofructokinase (EC:2.7.1.11), existing as a homotetramer in bacteria and mammals, has two isozymes in *Escherichia coli *and related species (pfkA and pfkB). pfkB is a minor phosphofructokinase which is not evolutionary related to the major isozyme (gene pfkA). In Figure 7, *Shigella flexneri 8401 *has no phophofructokinase gene and BAG clustering clearly divides class I and class II isozymes. To detect two isozymes classes in *Shigella flexneri 8401*, two sequential CSR-HMM searches detect SFV_3578 (class I) and SFV_1498 (class II).

### Step 3: Evaluation of enzyme classification

Candidate enzymes predicted by sequence similarity often need to be examined further. ComPath provides several computational methods for further evaluation of candidate matches. Phylogenetic tree analysis is probably the most powerful method that visualizes the relationship among candidates and known enzyme sequences. ComPath uses PHYLIP package [32] to generate a phylogenetic tree by using the neighbor-joining algorithm. The phylogenetic tree can be viewed by a web browser (in PNG format) or ATV trees viewer program (via Java applet) [33]. Multiple sequence alignment is also available to users while generating the phylogenetic tree. BAG sequence clustering program [26] is used for both the enzyme match and refinement step. Finally, Gibbs motif sampler [3] and iGibbs [34], our in-house motif detection algorithm, can be used to predict conserved regions and motifs in a set of enzyme sequences. Motif information from CDD [20] and PROSITE [14] are also provided.

Context-based sequence analysis techniques generally improve accuracy of gene function prediction made by using traditional sequence similarity-based search methods. The context involves a group of genes physically (e.g., gene neighbors on the chromosomes) or functionally (e.g., interacting proteins or those involved in the same pathway) related to the gene being analyzed. In particular, there is growing evidence that evolutionary relationships of multiple genomes can be understood better when metabolic pathways and functional context can be enforced by detailed reconstruction of relevant metabolic or other cellular pathways [35,36].

## Results and Discussion

### Empirical evaluation of enzyme prediction methods

**Figure 8:**
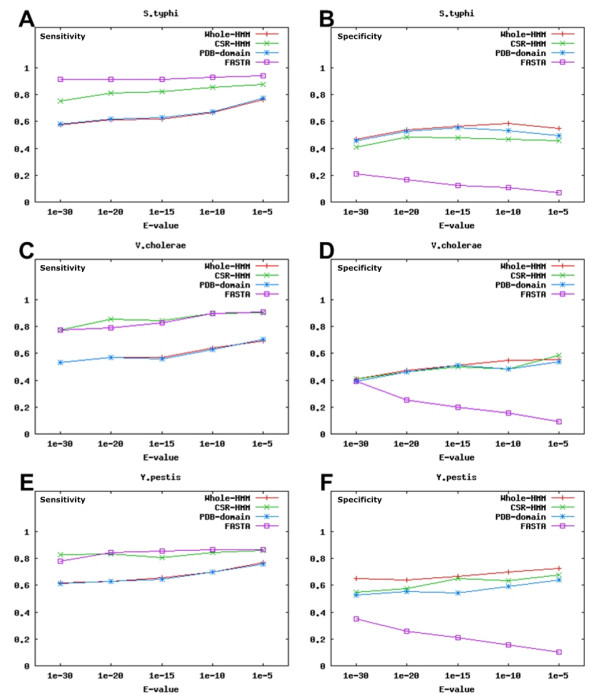
**Comparison of four methods used in ComPath**. The performance comparison of Whole-HMM search, CSR-HMM search, PDB-domain search, and FASTA search methods in terms of sensitivity and specificity. Glucolysis/Gluconeogenesis pathway (Pathway ID: 00010) and five reference genomes were selected: *Escherichia coli K12 *(NC_00913), *Haemophilus influenzae *(NC_000907), and *Salmonella typhimurium LT2 *(NC_003197) from *Proteobacteria; *and *Bacillus anthracis str. Ames *(NC_003997) and *Bacillus halodurans C-125 *(NC_002570) from *Firmicutes*. Three well-annotated genomes (*Salmonella enterica subsp. enterica serovar Typhi Ty2*,, *Vibrio cholerae O1 biovar eltor str. N16961 chromosome II *(NC_002506), and *Yersinia pestis CO92 *(NC_003134) were selected as query genomes to accurately evaluate performance of the four prediction methods. The plots show sensitivity and specificity of the four methods. See also Table 2.

Figure 8 and Table 2 shows our experiment data. To compare the performance of four gene search methods (Whole-HMM search, CSR-HMM search, PDB-domain search, and FASTA search) used in ComPath, we selected the Glucolysis/Gluconeogenesis pathway (Pathway ID: 00010) and five reference genomes: *Escherichia coli K12 *(NC_00913), *Haemophilus influenzae *(NC_000907), and *Salmonella typhimurium LT2 *(NC_003197) from *Proteobacteria*; *Bacillus anthracis str. Ames *(NC_003997) and *Bacillus halodurans C-125 *(NC_002570) from *Firmicutes*.

**Table 2:** Sensitivity and specificity data

**Species**	**Methods**	**E-value**	**Sensitivity**	**Specificity**
**S. typhi**	FASTA	1e-30	0.9145	0.2121
		1e-20	0.9153	0.1685
		1e-15	0.9153	0.1238
		1e-10	0.9310	0.1095
		1e-5	0.9391	0.0721
	Whole-HMM	1e-30	0.5726	0.4697
		1e-20	0.6102	0.5393
		1e-15	0.6187	0.5619
		1e-10	0.6638	0.5839
		1e-5	0.7652	0.5481
	CSR-HMM	1e-30	0.7521	0.4091
		1e-20	0.8136	0.4831
		1e-15	0.822	0.4762
		1e-10	0.8534	0.4672
		1e-5	0.8783	0.4567
	PDF-domain	1e-30	0.5812	0.4545
		1e-20	0.6186	0.5281
		1e-15	0.6271	0.5524
		1e-10	0.6724	0.5328
		1e-5	0.7739	0.4952
**V. cholerae**	FASTA	1e-30	0.7722	0.3934
		1e-20	0.7901	0.25
		1e-15	0.8293	0.2
		1e-10	0.8974	0.1532
		1e-5	0.9103	0.0914
	Whole-HMM	1e-30	0.5316	0.401
		1e-20	0.568	0.475
		1e-15	0.561	0.51
		1e-10	0.628	0.5484
		1e-5	0.6923	0.5543
	CSR-HMM	1e-30	0.7722	0.401
		1e-20	0.8519	0.4625
		1e-15	0.8415	0.5
		1e-10	0.8974	0.4839
		1e-5	0.7051	0.5829
	PDF-domain	1e-30	0.5316	0.3934
		1e-20	0.5680	0.4625
		1e-15	0.561	0.51
		1e-10	0.6282	0.4839
**Y. pestis**	FASTA	1e-30	0.7805	0.35
		1e-20	0.8434	0.2553
		1e-15	0.8537	0.2105
		1e-10	0.8675	0.1579
		1e-5	0.8675	0.1
	Whole-HMM	1e-30	0.6098	0.65
		1e-20	0.6265	0.6383
		1e-15	0.6463	0.6667
		1e-10	0.6988	0.6974
		1e-5	0.7590	0.725
	CSR-HMM	1e-30	0.8293	0.55
		1e-20	0.8313	0.5745
		1e-15	0.8049	0.6491
		1e-10	0.8434	0.63158
		1e-5	0.759	0.675
	PDF-domain	1e-30	0.6098	0.525
		1e-20	0.6265	0.5532
		1e-15	0.6463	0.5439
		1e-10	0.6988	0.5921
		1e-5	0.759	0.6417

Then, three well-annotated query genomes (*Salmonella enterica subsp. enterica serovar Typhi Ty2 *(NC_004631), *Vibrio cholerae O1 biovar eltor str. N16961 chromosome II *(NC_002506), and *Yersinia pestis CO92 *(NC_003134) were selected as query genomes to test how correctly each search method detects metabolic pathway components (enzymes) using the "enzyme profile" of reference genomes. Sensitivity and specificity of each method were calculated after the enzyme profiles of each EC group were searched against these genomes.

If the EC number of a detected enzyme is the same as that of the enzyme profile, this enzyme was considered "true positive (TP)". "False positive (FP)" was the case that the EC numbers of the enzyme profile and detected enzyme were different (or no EC number was assigned to the enzyme). If an enzyme has a correct EC identifier, but was not detected, this enzyme was considered "false negative (FN)". Finally an enzyme is considered "true negative (TN) [36] if an enzyme was not detected and also has different (or no) EC number from the enzyme profile. Sensitivity (TP/(TP+FN)) and Specificity (TN/(TN+FP)) were calculated and the results plotted using GnuPlot 4.0.

According to our experiment, FASTA and CSR-HMM searches outperformed Whole-HMM and PDB-domain searches in terms of sensitivity, but FASTA search showed poor performance in terms of specificity, detecting more false positive as E-value increases. Overall, our CSR-HMM search method performed best in terms of sensitivity and specificity.

## Conclusion

We have developed a web-based comparative pathway analysis workbench for biologists or medical scientists. Using the spreadsheet-style interface, users can freely compare pathways in multiple genomes, predict new enzyme candidates using various sequence analysis techniques including our own CSR-HMM method, and refine the prediction result. In case a given pathway/subsystem is unique only to the query genome, comparative and pathway/subsystems-based genome annotation may not work. Fortunately, pathways/subsystems are partially or fully conserved among close species in general, so pathway/subsystems-based genome annotation can take advantage of co-evolution, co-occurrence, and co-regulation relationship of pathway/subsystem component genes of query and reference genomes. Once a 'model' pathway/subsystem can be correctly defined, it is relatively easy to reconstruct it in query genome by referring to reference genomes. It worth noting that 'model' pathway representation (e.g. KEGG map) is artificially defined. It is always possible that several alternative pathways or hidden hierarchical networks can be found when we see the whole network from many different viewpoints and this is why the global pathway network analysis is also required for pathway analysis. ComPath plans to include more resources and computational tools. Among them are Structure-Function Linkage Database (SFLD) [37] and ProRule [38]. We will include more genome context search methods, including in-house tools such as MCGS [22,39] and OperonViz [40]. These tools will be useful to explore which enzymes in a pathway appear in a physically clustered form [25].

## Availability and requirements

**Project home page**: 

**Operating system(s)**: Platform independent.

**Other requirements**: Mozilla-based web browser and Java Runtime Environment (JRE v1.4.0 or higher) are recommended.

**Licence**: ComPath is freely available to academic and non-academic users

**Any restrictions to use by non-academics**: None

## Authors' contributions

KC and SK participated in system and database design and drafted the manuscript. KC prepared the figures and performed system comparison and evaluation test. All authors have read and approved the final manuscript.
